# Comparison of Yeasts as Hosts for Recombinant Protein Production

**DOI:** 10.3390/microorganisms6020038

**Published:** 2018-04-29

**Authors:** Antonio Milton Vieira Gomes, Talita Souza Carmo, Lucas Silva Carvalho, Frederico Mendonça Bahia, Nádia Skorupa Parachin

**Affiliations:** Grupo Engenharia de Biocatalisadores, Departamento de Biologia Celular, Instituto de Ciências Biológicas Bloco K 1º andar, Universidade de Brasília, Campus Darcy Ribeiro, CEP 70.790-900 Brasília-DF, Brazil; miltonvieirag@gmail.com (A.M.V.G.); talitacarmo@gmail.com (T.S.C.); u.lucas@gmail.com (L.S.C.), fredericombahia@gmail.com (F.M.B.)

**Keywords:** recombinant protein, yeast, Saccharomyces cerevisiae, Kluyveromyces lactis, Yarrowia lipolytica, Komagataella phaffii

## Abstract

Recombinant protein production emerged in the early 1980s with the development of genetic engineering tools, which represented a compelling alternative to protein extraction from natural sources. Over the years, a high level of heterologous protein was made possible in a variety of hosts ranging from the bacteria *Escherichia coli* to mammalian cells. Recombinant protein importance is represented by its market size, which reached $1654 million in 2016 and is expected to reach $2850.5 million by 2022. Among the available hosts, yeasts have been used for producing a great variety of proteins applied to chemicals, fuels, food, and pharmaceuticals, being one of the most used hosts for recombinant production nowadays. Historically, *Saccharomyces cerevisiae* was the dominant yeast host for heterologous protein production. Lately, other yeasts such as *Komagataella* sp., *Kluyveromyces lactis*, and *Yarrowia lipolytica* have emerged as advantageous hosts. In this review, a comparative analysis is done listing the advantages and disadvantages of using each host regarding the availability of genetic tools, strategies for cultivation in bioreactors, and the main techniques utilized for protein purification. Finally, examples of each host will be discussed regarding the total amount of protein recovered and its bioactivity due to correct folding and glycosylation patterns.

## 1. Introduction

Recombinant protein production emerged in the early 1980s, aiming at overcoming the limitations imposed by extraction of natural sources. For example, the first commercial pharmaceuticals, human insulin and growth hormone, were initially produced using the bacteria *Escherichia coli* as the host. Later, human insulin became the first licensed drug created using genetic engineering techniques in 1982, as recently reviewed [1]. Nowadays, recombinant protein is considered a milestone for drug discovery. Its current market size can verify its relevance. A recent study done by MarketsandMarkets revealed that the so-called “protein expression market” was worth $1645.0 million in 2017 and expected to reach $2850.5 million by 2022 [2]. Moreover, it is reported that, despite its various uses in the food, detergent, paper, chemical, and cosmeceutical industries, it is pharmaceutical and biotechnology companies that hold the largest share market. Thus, drug discovery is one of the driven forces for the continuous growth of recombinant protein market. For example in 2011–2016, 62 new recombinant proteins were approved by regulatory agencies in USA [3]. 

The development of drug discovery requires the expression of various proteins. Moreover, academic purposes such as the establishment of crystal proteins also demand the expression of a large number of proteins. For that reason, high-throughput strategies are often pursued in various organisms, as previously described [4,5,6]. Among the different hosts that are utilized for recombinant protein production, the bacteria *Escherichia coli* is widely known as a preferential host due to the vast availability of genetic tools, rapid growth, and simple cultivation techniques. Nevertheless, depending on the properties of the desired protein, utilization of *E. coli* might be laborious or not even possible. This is the case when the recombinant protein needs to be glycosylated or chemically modified after translation, or requires proteolytic processing. Therefore, generally the enzymes purified from *E. coli* need to pass through an in vitro process for the insertion of post-translational modifications, which adds steps during protein synthesis, resulting in an even costlier process and a reduced yield of final recombinant protein. Thus, as an alternative to in vitro modification, eukaryotic hosts such as yeasts, filamentous fungi, insects, and plant and mammalian cell lines have been utilized [7,8,9,10,11]. Among them, yeasts combine the simplicity of a unicellular organism, having lower nutritional demands when compared to insect and mammalian cell lines, with the ability to realize most of the post-translational modifications required for a biologically active recombinant protein. Therefore, yeasts have been utilized for the production of various pharmaceutical proteins [11,12]. For example, the hormones insulin and glucagon are industrially produced using *Saccharomyces cerevisiae* as a host [12].

Historically, the yeast *S. cerevisiae* has primarily been utilized as a host for recombinant protein production. The advantages of using this yeast have been summarized previously [12,13], but it should be emphasized that it has more natural adaptability to the harsh industrial-scale conditions and an ability to correctly produce and secrete biologically active eukaryotic proteins. Various recombinant proteins using *S. cerevisiae* as a host have been marketed [13,14]. Nevertheless, in recent years, many proteins have become commercially available using other yeasts as hosts. Among them, *Pichia pastoris* gained attention for marketing novel recombinant therapeutics such as human insulin, human serum albumin, hepatitis B vaccine, interferon-alpha 2b, trypsin, and collagen, among others [15]. *P. pastoris* is an obligate aerobic yeast that can use methanol as a carbon source. This last characteristic allowed for the development of an expression system based on the utilization of the inducible AOX1 promoter. Nearly 10 years ago, *P. pastoris* were reclassified to *Komagataella phaffii* [16]; therefore, in this review, it will be the name given to this yeast. When compared to *S. cerevisiae*, *K. Phaffii* is known for giving higher recombinant titers since this yeast is Crabtree-negative and under respiratory conditions does not lose carbon by producing ethanol, which results in higher biomass formation and consequently more recombinant protein [11]. For that reason, *K. phaffii* has been extensively explored for recombinant protein production, as previously reviewed [17,18,19,20]. 

The yeast *Kluyveromyces lactis* is also a respiratory Crabtree-negative yeast. Industrially, *K. lactis* is known for secreting β-galactosidase, a protein used mainly in the food industry for making lactose-free products. The first recombinant protein produced using *K. lactis* as a host was the bovine chymosin and up to now at least 20 recombinant proteins with application in the food and pharmaceutical industries have been produced using *K. lactis* [21]. Besides being Crabtree-negative, another advantage of *K. lactis* over *S. cerevisiae* as a host is that it metabolizes hexoses via glycolysis and the pentose phosphate pathway. Recently, *K. lactis*, as a host for heterologous protein production, has been extensively reviewed regarding the availability of its genetic tools and cultivation techniques [21]. 

Among the “non-conventional” yeasts, *Yarrowia lipolytica* is known for its ability to use hydrocarbons such as paraffin and oils as a carbon source. Its applications on an industrial scale have recently been reviewed and range from single cell protein, pre- and probiotics, and bioremediation of oily wastewaters to host for the production of recombinant enzymes replacement therapies (ERTs) and the homologous lipase (YlLip2p) that will be marketed for treating exocrine pancreatic insufficiency [22,23,24]. Moreover, it has been reported to secrete high levels of native and heterologous proteins. For example, wild-type strains of *Y. lipolytica* can emit 1–2 g/L of alkaline extracellular protease (XPR2) [25].

**Figure 1:**
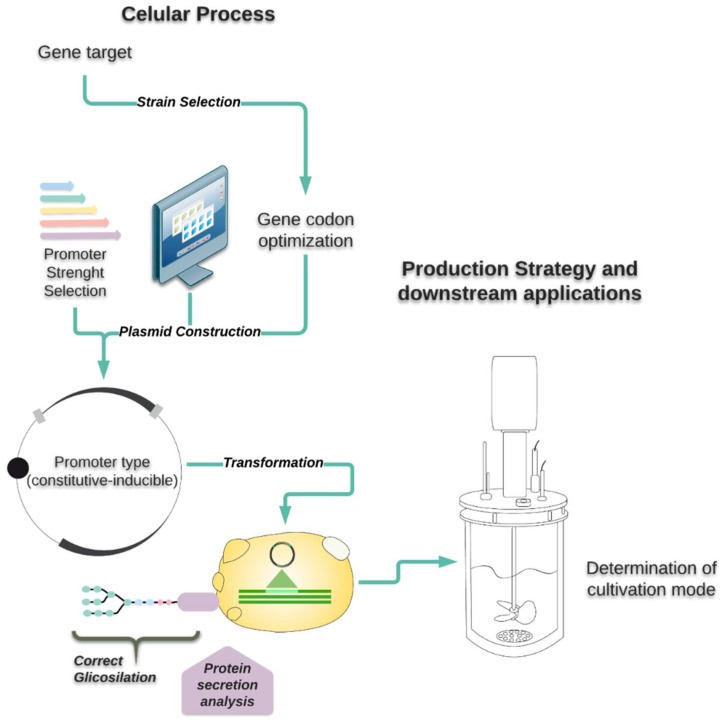
Main aspects to be considered for recombinant protein production in yeasts.

All the yeasts mentioned above have been extensively reviewed regarding the availability of genetic tools, type of post-translational modifications, and cultivation techniques. Nevertheless, this is the first time that a comparative assessment has been done for *S. cerevisiae*, *K. phaffii*, *K. lactis*, and *Y. lipolytica* as hosts for recombinant protein production. Figure 1 summarizes the aspects that will be discussed in this review. 

## 2. Availability of Genetic Tools.

The availability of genetic tools plays a prominent role when choosing a host for heterologous production. The vast majority of yeast synthetic biology tools have been developed in *S. cerevisiae* due to its well-annotated genome, genetic tractability, and overall ease of use [26]. Engineering of non-conventional yeasts is hindered by a lack of advanced genome editing tools and an incomplete understanding of their genetics, metabolism, and cellular physiology. However, they can provide many potential advantages over *S. cerevisiae* concerning pathway requirements, desired product profile, and biology. In contrast to *S. cerevisiae*, these non-conventional yeasts are Crabtree-negative and favor respiration over fermentation, phenotypes that are particularly useful for protein production [27]. Thus, the increasing availability of high-quality yeast genome sequences, efficient vectors and transformation methods, and emerging synthetic tools are broadening manipulation and understanding of non-conventional yeasts [26,27].

### 2.1. DNA Assembly Methods

The first DNA assembly method developed and still the most utilized one is the 45-year-old restriction and ligation, though it can be time-consuming and technically challenging according to the size of the fragment to be cloned. Therefore, many new DNA assembly methods have been developed in the past decade. Those can be divided into four main groups: restriction enzyme-based methods (e.g., Bio-Brick, Golden Gate), in vivo (e.g., DNA assembler-yeast) and in vitro (e.g., Gibson Assembly, USER—uracil-specific excision reagent cloning) sequence homology-based methods, and bridging oligo-based approaches (e.g., LCR—ligase chain reaction), as recently reviewed [28]. The plasmid repository addgene summarizes the criteria that may be utilized for molecular cloning and offers collections of plasmids that may be used in the different cloning technologies [29]. In *S. cerevisiae* a wide range of in vivo homology-based DNA assembly tools were developed and implemented [30,31] since homologous recombination (HR) is the dominant DNA repair pathway in this yeast. The repair system via HR is not frequently used in other yeasts, where nonhomologous end-joining (NHEJ) is the preferred repair pathway, making in vitro assembly methods more suitable [28]. For example, GoldenPiCS is a modular Golden Gate-derived *K. phaffii* cloning system that is used for protein production or other applications where the integration of various DNA products is required [32]. It allows for the assembly of up to eight expression units on one plasmid, with the ability to use different characterized promoters and terminators for each expression unit. In-depth information on recent advances in DNA assembly methods can be found in a recent review [33]. Recently, the Golden Gate System was also implemented for *Y. lipolytica* using the insertion of three genes for carotenoid production as a proof of concept [34].

### 2.2. Genetic Elements Applied to Recombinant Protein Production

Due to their critical role in expression cassette design, promoters are likely the most characterized and engineered genetic part in many yeast systems [26]. Well-characterized constitutive or inducible promoters with strong transcriptional activity are used to achieve overproduction of recombinant protein [35]. The most frequently utilized promoters in yeasts are listed in Table 1. Constitutive promoters offer simplicity and relatively constant levels of expression, while inducible promoters are commonly used when separation of growth and production is desired, possibly preventing unintentional selection of more rapidly growing non-recombinant cells or for the production of toxic proteins. The strong constitutive TEF1 and GPD (glyceraldehyde 3-phosphate dehydrogenase, also known as TDH3) promoters have frequently been employed to direct high-level expression of heterologous genes in *S. cerevisiae* [36]. However, utilization of strong constitutive promoters might lead to lower secretion efficiency due to aggregation of misfolded proteins, as reported in the expression of insulin precursor and α-amylase in *S. cerevisiae* [37]. In this sense, the utilization of inducible promoters is advantageous since it allows us to control gene expression levels in the presence and concentration of its inductor molecule. In *S. cerevisiae* the galactose-induced GAL1 and GAL10 promoters are frequently used [38].

Heterologous protein production in *K. lactis*, like alpha-amylase [39] and interferon alpha A [40], has frequently relied on *S. cerevisiae* promoters such as pGAL1 or pPGK, showing a high level of promoter element transferability between *S. cerevisiae* and *K. lactis*. Furthermore, its endogenous LAC4 promoter has also been routinely used due to its inherent strength and 100-fold induction by lactose/galactose, like for the production of phospholipase A2 from *Lactobacillus casei* [41]. Strategies with the methylotrophic *K. phaffii* often rely on their substantial methanol inducible promoter pAOX1; to date more than 300 recombinant proteins have been produced using this promoter [18].

Regarding the yeast *Y. lipolytica*, it has been the subject of several promoter engineering efforts beginning with the strong XPR2 alkaline extracellular protease promoter pXPR2, resulting in strong hybrid promoters currently available for recombinant protein production [26]. Nevertheless, some studies have reported that the use of XPR2 promoter is not attractive for industrial production of recombinant protein because it is active only at pH greater than 6 and requires large amounts of peptone in the cultivation medium. In those cases the replacement of the XPR2 promoter by the Translation elongation factor-1a TEF promoter was shown to almost double the secretion of recombinant protein [42]. Recently, a novel inducible promoter from *Y. lipolytica* EYK1 gene has been isolated and characterized. It has been shown that EYK1 promoter results in low expression levels when the yeast is cultivated in the presence of glucose or glycerol, being induced by the presence of erythritol or erythrulose [43].

Overall, the choice of a weak promoter is not attractive because this results in low levels of transcription of the gene of interest and consequently low amounts of the recombinant protein. Likewise, choosing a strong promoter is not always recommended either since the large amount of transcripts of a gene of interest can cause stress in the cell if the protein product of this gene activates the Unfolded Protein Response (UPR), resulting in cell death. In this way, knowledge of protein toxicity for the host, demands on protein folding and protein size are fundamental for choosing a proper promoter. In this regard, there are currently studies of promoter libraries testing their “strength” for use in yeast expression systems. For example, studies with the promoters of the G3P (Glyceraldehyde 3-phosphate dehydrogenase), ICL1 (isocitrate lyase), POT1 (protection of telomere), POX1 (acyl-CoA oxidase), POX2 and POX5 genes in yeast *Y. lipolytica* [44], promoters of the ADH3 (alcohol dehydrogenase), AOX1 (alcohol oxidase 1) and GAP (Glyceraldehydes-3-phosphate dehydrogenase) genes in *K. phaffii* [45] and different variant sequences of the LAC4 promoter in *K. lactis* [46]. A similar study showed the use of seven different promoters (TEF1, PGK1, TPI1, HXT7, PYK1, ADH1 and TDH3) in the production of the reporter protein β-galactosidase in *S. cerevisiae* compared to the Gal1 and Gal10 galactose induced promoters [36]. During all concentrations of glucose tested the TEF (Transcriptional elongation factor EF-1a) promoter was considered the strongest and ADH one of the weakest, suggesting that the TEF promoter is not affected by any glucose level. The HXT7 (hexose transporter) promoter, although considered weak in the presence of glucose, is considered stronger than the TEF promoter when there is glucose deprivation. In general, during glucose deprivation, the TEF and HXT7 promoters are still stronger than the Gal1 and Gal10 inductive promoters in the presence of galactose [36]. Altogether these results also emphasize that the promoter choice have also to be linked to the process strategy as it is affected by the physiological state of the cell. More in-depth information about different promoters utilized in yeasts can be found in previous reviews [26,47,48].

Transcriptional terminators serve a mechanistic role in transcription, but also influence mRNA stability. However, the impact of terminators on mRNA abundance and protein output is often underappreciated relative to promoters. Most commonly used *S. cerevisiae* expression vectors use a small set of previously identified, non-optimal native terminators such as CYC1t or ADH1t. *S. cerevisiae* terminators show a high degree of transferability across all four non-conventional yeast hosts [26].

Selectable marker genes are employed for sorting successfully transformed colonies containing the exogenous DNA of interest and are typically either dominant, auxotrophic, or autoselective markers. Dominant markers include genes conferring resistance to copper or appropriate antibiotics, e.g., chloramphenicol, G418, hygromycin, and zeocin, as previously reviewed [49]. Those are the preferred choice when there isn’t an auxotrophic yeast strain, or a rich cultivation medium is utilized. Nevertheless, the disadvantages of choosing a dominant marker can be related to the degradation or inactivation of the antibiotic. Moreover, its use is costly when compared to other selection markers or undesirable in the case of recombinant proteins applied in the food and pharmaceutical industries. Auxotrophic markers (e.g., HIS3, HIS4, LEU2, LYS2, TRP1, URA3) are designed to complement a specific auxotrophic mutation in the host strain, whereas autoselection systems rely on the expression of vital activity in host strains lacking such an activity. Thus using this selection system the plasmid is maintained to ensure the yeast survival independent of its culture conditions. Examples of auto selection systems are URA3 in a ∆fur1 (uracil phosphoribosyl transferase) background and FBA1 (fructose biphosphate aldolase) in a ∆fba1 background [49]. For *S. cerevisiae* and *K. lactis*, the LEU2 gene and the G418 resistance gene are the most popular selection markers while for *Y. lipolytica* the LEU2 and URA3 genes are mainly used. In *K. phaffii* HIS4 and zeocin are most frequently employed.

Elimination of the selection marker may be done using the Cre–loxP system initially designed for *S. cerevisiae* [50] but already implemented for the non-conventional yeasts [51,52]. For this, the selection marker’s expression cassette is flanked by loxP sites and integrated into the genome. After selection of positive transformants, the Cre recombinase is added to the yeast strain enabling the excision of the selection cassette leaving behind a single loxP site at the original site of integration [53]. Other marker recycling systems like hisG and lacZ have been developed but are rarely used in yeasts since it requires a counter-selection such as growth on media supplemented with 5-fluoroorotic acid (5-FOA) for URA3 excision [27].

### 2.3. Vector Availability

Production of recombinant proteins in *S. cerevisiae* can be done using three types of vectors: integration plasmids (YIp), episomal plasmids (YEp), and centromeric plasmids (YCp) as recently reviewed [54]. YEps are plasmids based on the endogenous 2μ origin of replication maintained in high-copy number inside the cell (5–30 copies), which enables robust gene expression, but it can impose a substantial burden on cells resulting in increased plasmid instability. YCPs are plasmids based on a combination of autonomously replicating sequence (ARS) and yeast centromeric sequence (CEN) maintained in low-copy copy numbers (1–2 copies), which are more stable, but lower gene expression levels limit their use. Thus integration of an expression cassette in a target locus on a native yeast chromosome is beneficial because it allows the removal of the selective pressure after the recombinant strain is constructed.

In non-conventional yeasts, plasmids options are more limited. While functional plasmids are available for the three non-conventional yeasts, they tend to be low copy number and show variable expression across cells in a single population [27]. There are functional 2μ-based plasmids in *K. lactis*, but those have been reported to be much less stable than in *S. cerevisiae*. Hence, genomic integration is still preferred [26]. In *K. phaffii*, homologous integration of expression cassettes occurs with only 1 to 30% of success rate, even when extended homologous overhangs (1 kb) are used. Whereas in *S. cerevisiae* the rate is approximately 100% for short 50 bp homologous arms, suggesting a stronger role played by NHEJ in the former when compared to the latter, where HR occurs near exclusively [55].

Frequently, multiple integrations of an expression cassette are desired to increase the total amount of the recombinant protein. In *S. cerevisiae*, 5–7 copies of human alpha-fetoprotein genes were integrated into the chromosome resulting in the successful secretion of human alpha-fetoprotein to the culture medium [56]. Nevertheless, when the integration of the expression cassette is random, it can result in unwanted disruptions of open reading frames or other genomic elements. Also, expression of heterologous genes has been shown to be highly dependent on the integration site. Therefore random integration can result in variable expression across transformants [27,57]. A recent study showed a 25-fold range variation of eGFP reporter protein fluorescence on non-specifically integrated *K. phaffii* transformants [55]. In *Y. lipolytica*, copy number and gene expression were improved by 80% by fusing different promoters upstream of the centromeric region on the available *Y. lipolytica* CEN plasmid [58]. Furthermore in *Y. lipolytica*, there is a family of vectors having either TR *zeta* or rDNA fragment for multicopy integration using the ura3d4 allele as auxotrophic marker and the strong inducible promoters pICL1 or pXPR2 [59]. Successful strategies have been implemented to enhance HR in non-conventional yeasts such as disruption of KU70, and KU80 genes that are essential for NHEJ have shown to increase the efficiency of homologous recombination up to 97% *K. lactis* [60] and 90% in *K. phaffii* [61]. In *Y. lipolytica* the KU070 gene was replaced by the hygromicin dominant marker and used for development of various auxotrophic strains [62]. Moreover the addition of hydroxyurea to cultures under exponential growth of *Y. lipolytica*, *K. lactis*, and *K. phaffii* have shown to increase homologous recombination by arresting S-phase of yeast cell cycle [63].

### 2.4. Genome Editing Techniques

As mentioned before, integration of heterologous genes into the host chromosomes reduces metabolic burden and provides genetic stability, resulting in recombinant organisms more suitable to be scaled up. To increase genome integration events, endonucleases that introduce site-specific double-stranded breaks (DSBs) have recently been employed. Due to deleterious effects of DSBs, repair pathways are activated to eliminate the double genome break. This event is favored when a template DNA is supplied to the microorganism host. Thus the high integration efficiencies and ease of use provided by these nucleases enable marker-less and precise integration in a wide range of cell types and organisms, including yeasts [64,65].

The most commonly known custom hybrid nucleases are Zinc Finger Nucleases (ZFNs), Transcription Activator-Like Effector Nucleases (TALENs) and CRISPR associated nuclease 9 [66]. Since the first two are rarely utilized in yeasts and have not been used for increased recombinant protein production, they will not be discussed here. The Clustered Regularly Interspaced Short Palindromic Repeats and its Associated nuclease 9, CRISPR/CAS9, have revolutionized the gene manipulation for the various applications due to its higher specificity and availability of genetic tools and protocols when compared to ZFNs and TALENs. Most CRISPR/Cas9 systems require only two components, namely a guide RNA (gRNA) complementary to the targeted region in the genome and an RNA guided DNA endonuclease (e.g., Cas9). Briefly, in this system, the endonuclease Cas9 binds a guide RNA molecule (gRNA) that targets a sequence-specific site in the genome. The Cas9-gRNA complex then induces a DSB, which can be repaired through NHEJ or HR, depending on the presence of a donor DNA with homology arms and the predominant repair mechanism of the host cell [66]. The fundamental difference between natural occurring HR and CRISPR/Cas9 mediated HR is that natural HR repair relies on a strand break that occurs coincidentally at the target locus, whereas a DSB is artificially created when using CRISPR/Cas9 [65]. This system has been implemented in *S. cerevisiae*, *K. lactis*, *K. phaffii* and *Y. lipolytica* [65,67,68,69,70,71] although no direct effect has associated with recombinant protein production up to this date. In *S. cerevisiae*, utilization of CRISPR/CAS9 has shown to increase recombination efficiency and feasibility for in site-specific mutation and allelic replacement [67]. In *K. lactis*, near-perfect targeting (≥96%) and HR-mediated repair DNA integration occurred at practicable rates in strain CBS 2359 (31%) origin of replication and two constitutive expression cassettes, for Cas9 and a ribozyme-flanked gRNAs [69]. More recently the utilization of CRISPR/Cas9 system in a *K. phaffii* strain that had KU07 deleted have shown overcome the drawback of low-frequency integration resulting inefficiencies of approximately five times higher when compared to the utilization of CRISPR/CAS9 in a strain with intact KU070 [68].

## 3. Post-Translational Modifications Related to Bioactive Recombinant Proteins

Post-translational modifications involve any process that modifies the protein composition. Examples of post-translational modifications (PTMs) consist of the reversible addition of a chemical group like phosphate, carbohydrates in glycosylation, and polypeptides in ubiquitylation. Additionally, PTM may also englobe proteolytic processes during protein maturation or even the modification of amino acids such as deamination processes [72]. Those modifications are often related to the recombinant protein activity and its direction inside the cell. For instance, misleading during PTM modifications may result in miss location or even protein degradation by the ubiquitination pathway. Thus the relevance of PTM modification reflects in protein folding and consequently on its biological activity. For instance, PTMs that are made differently from the natural such as glycosylation with more or fewer sugar chains than expected may prevent the protein from adequately folding. An example of the importance of a glycosylation process in the production of recombinant protein is the production of the recombinant elastase (rPAE) protein in *Komagataella phaffii*. The *N*-glycosylation process, for example, assists the proteins along the way to proper folding and the addition of an *N*-glycosylation site on the elastin (rPAE) propeptide at the N51 or N93 positions stimulated the increase in rPAE production by 104% or 57%, respectively [73].

In yeasts, the most common PTMs include acetylation, amidation, hydroxylation, methylation, N-linked glycosylation, O-linked glycosylation, phosphorylation, pyrrolidone carboxylic acid, sulfation, and ubiquitylation. Yeasts can make the most ordinary PTMs without significant differences from the pattern founded in mammalian cells. Nevertheless, glycosylation is one of the leading challenges when producing recombinant proteins in yeasts. Since approximately 60% of recombinant proteins approved for therapeutic use are glycoproteins, and an annual growth rate of this class of proteins is estimated at 26%, it is fundamental to have an adequate glycosylation pattern when using yeasts as hosts [3].

**Figure 2:**
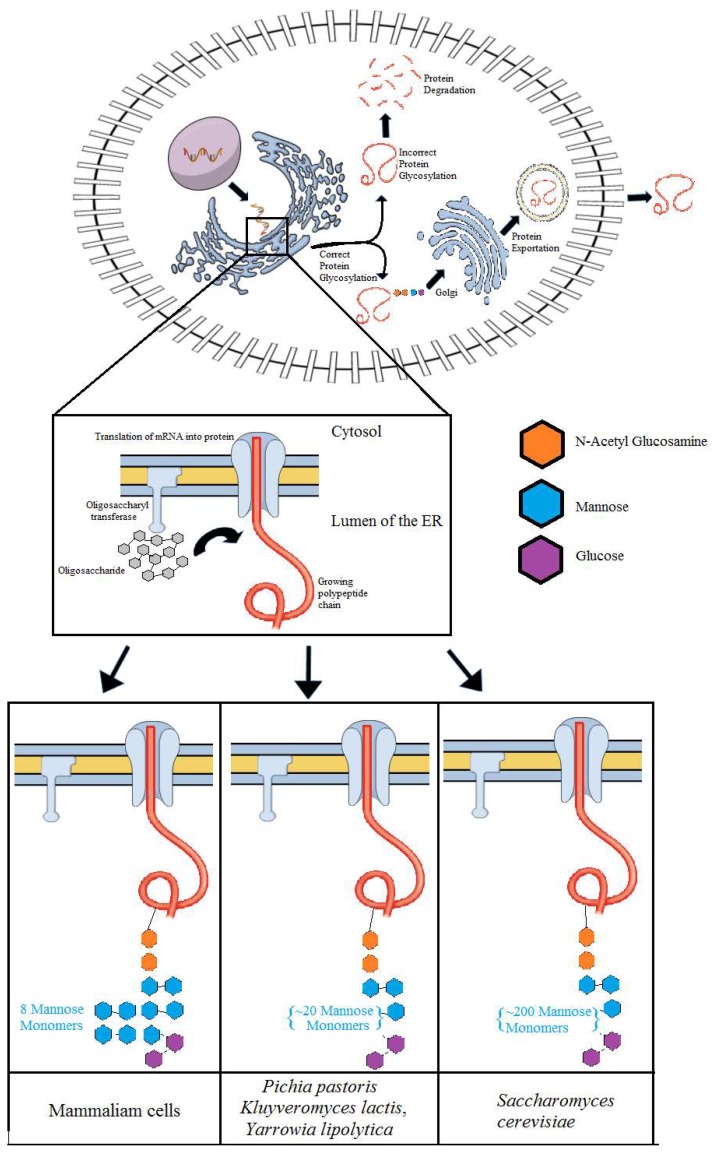
Recombinant protein glycosylation pattern in yeasts and mammalian cells.

Yeasts have a conserved glycosylation system in which a 14-sugar oligosaccharide is transferred to the nascent proteins in the membrane of endoplasmic reticulum (ER) by the action of enzymes named oligosaccharyltransferases (OST) (Figure 2). The oligosaccharide is attached to an asparagine residue of the peptide and serves as a signal to the yeast as to whether or not the protein can exit the ER [74]. Another transferase, called UDP-glucose: glycoprotein glucosyltransferase (UGGT), is also involved in the glycosylation process and transfers a glucose monomer as a signal in case the protein is not ready to exit the ER [75].

The glycosylation process in recombinant proteins depends on the amount and type of carbohydrate monomer that is added to the protein. Therefore, previous studies have shown that not only the glycosylation process but its kind may also result in miss activity of recombinant proteins. As an example, the human erythropoietin (EPO), a glycoprotein used in the treatment of anemia contains three N-glycans. This N-glycans, when removed, results in a protein with a reduced half-life and virtually no function in vivo [76]. Another enzyme, named glucocerebrosidase, is used in the treatment of Gaucher disease [77,78]. This protein needs mannose residues to keep its biological activity. Thus, the effect of glycosylation has been tested in different hosts [77,78]. In *S. cerevisiae*, besides mannose residues, there are other sugars that impair the bioactivity of the recombinant protein. In this case, the removal of non-mannose sugars is performed in the recombinant glucocerebrosidase prior to it being commercialized through costly in vitro processes [78]. Therefore, *E. coli* was considered a more suitable host for the recombinant production of recombinant glucocerebrosidase [77,78]. Specifically, *S. cerevisiae* is known for adding up to 200 mannose residues resulting in the hyper glycosylation of the recombinant protein [79]⁠. This type of modification can result in protein misfolding and prevent its exit from the ER, which in turns results in the loss of production titers. For example, it has been previously shown that the deletion of the gene responsible for the production of the enzymes mannosyltransferases increased the secretion of the β-glucosidase from *Saccharomycopsis fibuligera*, endoglucanase from *Clostridium thermocellum* and cellobiohydrolase from *Trichoderma reesei* to 156%, 105%, and 230%, respectively, compared with the control strain without gene deletion [80].

When compared to *S. cerevisiae*, *K. phaffii* is advantageous for adding mannose residues to a lesser extend (Figure 2) [81]. This could be verified with the enzyme glucoamylase from *Aspergillus awamori* that was heterologously produced using both *S. cerevisiae* and *K. phaffii* [82,83]. In both hosts, the recombinant protein was overglycosylated, presenting a molecular weight higher than the original enzyme by 23% and 13% in *S. cerevisiae* and *K. phaffii*, respectively. Nevertheless, this yeast may add mannose residues in recombinant proteins that are not naturally glycosylated. For example, the recombinant insulin-like growth factor I (IGF-I) that is not typically glycosylated is glycosylated in approximately 15% of the total protein secreted from *K. phaffii* [84]. Like *S. cerevisiae*, *K. phaffii* dos not have the ability to add the variety of sugars that are inserted in mammalian cells, carrying out the addition of mannose residues. 

In *Y. lipolytica* the endoprotease encoded by the XPR6 gene was initially shown to act on the secretion of the alkaline extracellular protease (AEP) protein encoded by the *XPR2* gene. This gene has a sequence containing secretion signals and a pro region that is cleaved by the endoprotease encoded by the *XPR6* gene, releasing the mature form of the AEP protein [85]. In addition, in the AEP encoding gene sequence there is also a pre region, which is necessary for its correct folding and also involved in a glycosylation process that is necessary for proper AEP secretion [86,87]. Other studies of lipase secretion in *Y.lipolytica* also directly related the deletion of the *XPR6* gene to the decrease in extracellular lipase activity by at least 75%, confirming that this gene is involved in the secretion of proteins other than AEP [88]. In addition, other studies have demonstrated the importance of the glycosylation pattern in the lipase Lip2 protein secreted by *Y. lipolytica* [89]. Substitution of amino acids present at the two glycosylation sites in Lip2 have shown that even with one or two mutations that avoid the glycosylation process, Lip2 continued to be secreted into the culture medium, suggesting that glycosylation does not affect the secretion process, but affects at different levels the activity of the enzyme depending on the TG substrate that is used in the enzyme assay of Lip2 [89].

Similar to the AEP protein, in *Y. lipolytica* the lipase Lip2 protein is also encoded from the *LIP2* gene, generating a polypeptide chain containing: (a) a pre-sequence signal peptide containing 13 amino acids; (b) a sequence denominated dp containing four dipeptides that are used as sites for processing by a diamino peptidase; (c) a pro sequence of 12 amino acids containing a site for processing by the endoprotease encoded by the *XPR6* gene and the (d) 301 amino acid sequence of the lipase. Although the secretion signal of the AEP protein (XPR2pre) is the most commonly used sequence to produce heterologous proteins in *Y. lipolytica* [24], the Lip2 protein also naturally has a secretion signal called LIPpre; a comparison of this peptide signal with others in the secretion of recombinant proteins has been made previously [24]. Recently, one of these studies tested different combinations of the pre, dp, and pro regions of the secretion signals XPR2 and LIP during the secretion of the lipase protein [90]. From the comparison of *Y. lipolytica* strains containing the same promoters, terminators, replication vectors, and selection markers, the XPR2 secretion signal was shown to be more effective than the LIP secretion signal [90]. The highest activity of lipase secretion was found in the strain containing a truncated version of the lipase lacking the dp region and containing the XPR secretion signal (XRP2pre-XRP2pro-Lipase), whereas lipase secretion activity containing the lip secretion signal (LIPpre-dp-LIPpro-Lipase) presented approximately 14% of that value. Strains guided by the XPR secretion signal containing the dp region (XRP2pre-dp-XPRpro-Lipase) and without the pro and dp regions (XRP2pre-Lipase) presented activities, respectively, 74% and 63% of the highest activity (XRP2pre-XRP2pro-Lipase) (Table 2).

To overcome the type and extent of glycosylation, different studies have reported the genetic modification of *K. phaffii* to create a glycosylation pattern similar to mammalian hosts [91,92]. For example, in the production of the human protein midkine, the amino acid residues involved in receiving *O*-glycosylation have been replaced by other amino acids, preventing the *O*-glycosylation of these residues in the protein and resulting in an active and non-immunogenic protein [93]. Another example is the deletion of genes involved in glycosylation, which resulted in the reduction of the glycosylation of the recombinant protein human antithrombin to a level of 1%, whereas in the yeast with the gene not deleted, the levels were 20%. Unlike the antithrombin from wild yeast, the antithrombin produced on the “humanized” yeast with the deleted gene was not immunogenic [94].

While there are extensive studies about glycosylation patterns in *S. cerevisiae* and *K. phaffii*, less information is available regarding glycosylation patterns in *K. lactis* [95,96,97]. Nevertheless, it is known that in *K. lactis*, the *N*-glycosylation process results in the addition of approximately 30 mannose residues (Man(>30)GlcNAc(2)) (Figure 2). Therefore, recombinant proteins are less immunogenic when *K. lactis* is used as a host when compared to *S. cerevisiae*. Explicitly, a study has shown that disruption of OCH1 that encodes for an α-1,6-mannosyltransferase reduced the glycosylation size of the recombinant Granulocyte Macrophage Colony Stimulating Factor (GM-CSF) protein from Man(>30)GlcNAc(2) to Man(13-14)GlcNAc(2) [97]. An additional deletion of the gene MNN1 that encodes for an α-1,3-mannosyltransferase reduced the mannose residues to nine. Altogether the results obtained confirmed the role of the two mannosyltransferases in the *N*-glycosylation process in *K. lactis* [97]. *Y. lipolytica* has the same characteristics of PTMs and glycosylation found in *K. lactis* and *K. phaffii*. Among the yeasts described here, this one has been the subject of fewer studies regarding its glycosylation mechanism. Similar to *K. lactis*, the genes OCH1 and MNN9, homologous to MNN1, were deleted in *Y. lipolytica*, resulting in fewer mannose residues in the recombinant glucocerebrosidade when compared to the same recombinant protein produced using the wild-type strain [98].

In addition to the glycosylation pattern described in yeasts, chaperones are known as helper proteins, responsible for directing protein folding. This is a fundamental step in the protein maturation process since protein misfolding leads to its accumulation in ER and thus reduces the amount of secreted protein [99]. Among different chaperones, the protein disulfide isomerase (PDI) is known to catalyze the formation of disulfide bonds between cysteine residues of a given protein [100]. In *K. phaffii*, co-production of PDI and the recombinant Interleukin-1 receptor antagonist (IL1ra) resulted in a three-fold increase in protein secretion when compared to the strain only producing IL1ra [101]. In another study, co-production of PDI and the recombinant *Necator americanus* secretory protein (Na-ASP1), which harbors 20 cysteines, have also shown increased secretion levels of the recombinant protein when an increased copy number of PDI encoding gene was used [102].

Besides PDI, heat shock proteins (Hsp) are among the main chaperones described to contribute to protein folding. Such proteins are divided into families like Hsp60 and Hsp70. Briefly, Hsp60 and Hsp70 have an affinity for exposed hydrophobic regions of proteins binding to them by the hydrolysis of ATP in ADP. This results in repositioning of the hydrophobic region and thus directing the correct protein folding [103]. Among several chaperones, an HSP70 called binding protein (BiP) is present in yeasts and has been known to contribute to protein folding in the ER [104]. In *S. cerevisiae*, BiP levels were adjusted to the range of 5–250% and tested in co-expression of three recombinant proteins [105]. A study has shown that reduction of BIP levels reduced the total amount of secreted protein. Nevertheless, increased BiP levels did not show any effect of protein secretion levels [105].

Also, the gene encoding for BiP, kar2 in yeast has been associated with the unfolded protein response (UPR), as previously reviewed [99,106]. UPR is activated at a transcriptional level when BiP is bound to unfolded proteins. This leads to an increased level of the transcription factor HAC1, which activates UPR at the RNA level [99,106]. In both *S. cerevisiae* and *K. phaffii*, the production of recombinant proteins was shown to trigger UPR response mediated by HAC1 levels [107]. For example, in *S. cerevisiae* the relationship between the deletion of the HAC1 gene and the production of the recombinant α-amylase from *Bacillus amyloliquefaciens* and endoglucanase EGI from *Trichoderma reesei* was investigated [108]. The results showed that secretion of α-amylase and endoglucanase decreased by about 70% and 40%, respectively, when HAC1 was deleted. When HAC1 was overexpressed, this resulted in an increase by about 140% in the secretion of the recombinant α-amylase [108].

## 4. Yeast Secretion Factors

Secretion factors are peptide signals located in the N-terminal of a protein. They are responsible for guiding the recombinant protein trough the Golgi complex to the extracellular environment. In yeasts, the most utilized secretion factor is the α–mating factor (MF) peptide. This peptide is composed of two sequences, pre- and pro, containing 19 and 67 amino acid residues, respectively. The pro sequence also has three N-linked glycosylation sites and a Kex2 endopeptidase site [109]. In the ER, the α-mating factor secretion signal occurs in the following steps: (1) peptidases remove the pre-sequence, allowing the Ste13 dipeptidase to eliminate the (2) cleavage of the pro sequence by Kex2 endopeptidase and finally two Glu-Ala dipeptides in the Golgi [110]. The utilization of α-MF leader resulted in different secretion levels described for *S. cerevisiae*, *P. pastoris*, *Y. lipolytica*, and *K. lactis*, as shown in Table 2. Moreover, the modification of the secretion factor using genetic engineering, directed evolution, or codon optimization has been previously studied, aiming at increased secretion titers of the recombinant protein in *K. phaffii* [111]. It had been shown that mutations of α-MF secretion factor in amino acids 57–70 increased the secretion of the recombinant proteins horseradish peroxidase and lipase by at least 50% [111].

In *K. lactis*, besides the utilization of the α–MF leader sequence from *S. cerevisiae*, other secretion signals such as the ones from the genes SUC2, KT, and KI α-factor have also been utilized for recombinant protein secretion. Its use and relation to the recombinant protein secreted are shown in Table 2. In *K. phaffii* there are commercially available vectors that offer eight different secretion signals: α-amylase signal sequence from *Aspergillus niger*, Glucoamylase signal sequence from *Aspergillus awamori*, Serum albumin signal sequence from *Homo sapiens*, Inulinase pre sequence from *Kluyveromyces maxianus*, Lysozyme signal sequence from *Gallus gallus*, and Invertase signal sequence, Killer Protein signal. In *Y. lipolytica* the secretion of a protein called alkaline extracellular protease (AEP), encoded by the XPR2 gene, resulted in the isolation of a pre-/pro region that is used for recombinant protein secretion in this yeast (Table 2). Moreover, unlike some other secretion sequences, the XPR2 pro region acts as a protective peptide ensuring a correct folding and transport of the protein by the cell [86].

A previous study compared the secretion capacity of six extracellular proteins (Cellulase I and II, Galactanase I, Xylanase I, Polygalacturonase I and Lipase I) in five yeasts (*Saccharomyces cerevisiae*, *Hansenula polymorpha*, *Kluyveromyces lactis*, *Schizosaccharomyces pombe*, and *Yarrowia lipolytica*), three of which are explored in this review [114]. As expected, the results showed that *S. cerevisiae* has a secretion capacity lower than *K. lactis* and *Y. lipolytica*. This has been attributed to the fact that *S. cerevisiae* evolved in a brewer environment, where protein secretion requirements are minimal. Although all yeasts have been able to produce proteins in their active end forms, the secretion activity per cell of the cellulase II was 44-fold higher in *Y. lipolytica* and 10-fold higher in *K. lactis* compared with *S. cerevisiae.* Similarly, the secretion activity per cell of the lipase I was 10-fold higher in *Y. lipolytica* and without differences in *K. lactis* compared with *S. cerevisiae*. Finally, it has been shown that *Y. lipolytica* is one of the best alternatives for *S. cerevisiae* regarding secretion levels since it was shown to have a secretory capacity 40 times higher than *S. cerevisiae* [114].

## 5. Cultivation Strategies for Maximization of Recombinant Proteins in Bioreactors

Variations in cultivation strategies among the different yeasts will include oxygen requirement and co-product formation according to each yeast type of metabolism. For example, for being a fermentative yeast, optimization conditions of recombinant proteins in *S. cerevisiae* involves the ability to use different sugars and the relation between pH and percentage of dissolved oxygen (DO), while in *K. phaffii* oxygen is always present and may be used as a criterion for process optimization [124]. Moreover, pH, osmolarity, and temperature have previously been shown to influence recombinant protein production [125,126]. For example reduction of cultivation, temperature resulted in a 3-fold increase in the specific productivity of two different secreted Fab antibody fragments in glucose-based bioreactor cultivations [127].

**Figure 3:**
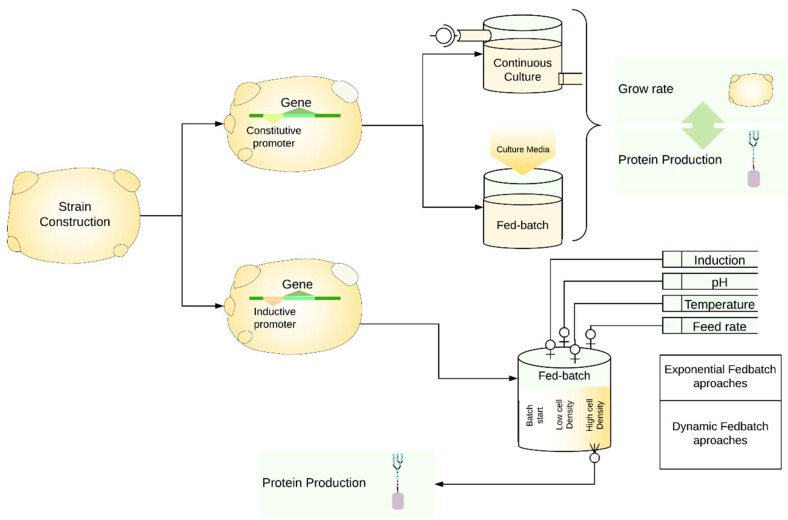
Cultivation strategies choice based on the promoter utilized for recombinant gene expression.

Several process parameters that affect recombinant protein production in yeasts have been previously studied [19,128,129,130,131,132]. Process optimization is primarily done in *K. phaffii*, which reflects the vast number of publications over the last 10 years. The same is not observed for *S. cerevisiae*, *K. lactis*, and *Y. lipolytica*, where the number of studies regarding process optimization for recombinant protein production is limited. Nevertheless, independent of chosen yeast, optimization of cultivation conditions depends on how the recombinant strain is constructed (Figure 3).

### 5.1. Recombinant Genes under the Control of Constitutive Promoters

In case a constitutive promoter is utilized, fed-batch or continuous cultivation may be applied whereby the specific growth rate (μ) will be adjusted to maximize recombinant protein production. For example, continuous glucose feeding was employed to optimize the recombinant production of lipase LIP2 (YlLIP2) from *Y. lipolytica* resulting in 13,500 U/mL and 120 g DCW/L YlLIP2 activity and cell density in a 10-L scale bioreactor [133]. Among the different feeding strategies, exponential feeding is considered an effective method to reach maximum cell growth and consequently maximum recombinant protein production since it has been reported that protein productivity is proportional to higher growth rates [134]. The same survey also stated that the exponential feed strategy should be performed after the selection of suitable carbon source; determination of average and maximum specific growth rate and biomass yield on substrate and evaluation of the effect of specific growth rate on protein production rate [134].

Another thing that was shown to be fundamental for process optimization is ensuring that, besides the limiting substrate in the feed, no other nutrient is limited during recombinant protein production. Recently, a transcriptional study done in *K. phaffii* cells producing the recombinant porcine carboxypeptidase B (CpB) employed fed-batch cultivation using either methanol or glucose as the carbon source [135]. As the aim of the study was to identify macronutrient limitations in those carbon sources, the recombinant gene was cloned under the pGAP promoter. Nutrients such as sulfate and nitrogen were shown to be limiting in both glucose and methanol, whereas the limitation was higher in the following carbon source. Thus fed-batch cultivations were performed with the addition of (NH_4_)_2_SO_4_ and (NH_4_)_2_PO_4_, which resulted in 52% and 60% higher recombinant CpB activity [135].

In *S. cerevisiae*, the effect of a specific growth rate on the recombinant production of α-amylase and human insulin was evaluated using carbon-limited chemostat [136]. Productivities of α-amylase and human insulin were achieved at dilution rates of 0.05 h^−1^ and 0.2 h^−1^, respectively. Surprisingly, quantitative PCR revealed that only the transcript levels of recombinant insulin were increased proportionally to increased dilution rates [136]. 

### 5.2. Recombinant Genes under the Control of Inducible Promoters

When the recombinant gene is cloned under the regulation of an inducible promoter, fed-batch strategies are preferentially used. In *K. lactis* fed-batch strategy achieved the highest protein titers on an industrial scale [21]. Fed batches for optimization of recombinant protein production are performed in a two-stage process where high cell density is desired prior to induction of heterologous gene transcription (Figure 2) [128,132,137].

In *K. phaffii* the most common strategy is to clone the heterologous gene under the control of an AOX promoter that is inducible in the presence of methanol. In this case, glycerol is commonly used to produce sufficient biomass before methanol induction. The utilization of glycerol pre-induction has been found to be beneficial for recombinant protein production [138]. The higher levels of UPR-related proteins in the glycerol batch seem to prepare the cells for efficient secretion in the methanol induction phase [138]. Generally, after the batch phase, a short transitional period is required to deplete the residual glycerol and eliminate possible repression in the induction phase. Next, methanol is added as the sole carbon source to induce recombinant gene expression. The ability to utilize methanol as a carbon source and inductor of gene expression is considered critical to the success of *K. phaffii* as a host for recombinant protein production since it has been shown that in this yeast transcriptional regulation has a stronger influence than translational regulation on recombinant protein production [139].

When a plasmid containing an expression cassette based on pAOX is inserted in a *K. phaffii* strain, two phenotypes may be obtained: the methanol utilizing plus strain, MUT^+^, meaning that the strain can use methanol as a carbon source in regular rates and the MUT^s^, meaning that the strain can utilize methanol but at slower rates. What defines whether the strain is MUT^+^ or MUT^S^ is the presence of one versus two copies of the AOX gene intact according to the plasmid integration into the yeast genome. Since the capacity of the strain to grow in methanol is dependent on its ability to utilize it, it is fundamental to determine the strain phenotype regarding methanol utilization. Besides Mut+ and MUT^s^, strains have different feeding strategies that affect the final recombinant protein production [140]. MUT^+^ strains have a specific growth rate on methanol of 0.15 h^−1^, while MUT^s^ growth is at the maximum rate of 0.03 h^−1^. The advantage of establishing a feeding strategy for a MUT^s^ strain is that even if the methanol concentration is increased in the bioreactor, the recombinant strain will not be able to utilize it, which facilitates the growth rate control [140]. Overall feeding strategies have been optimized for allowing growth rate in the range of 0.03–0.08 h^−1^, which can achieve a recombinant protein production rate up to 0.69 mg·g·h^−1^ [19].

Different studies about flux analysis during recombinant protein production and its relation to carbon source utilization suggested that utilization of a mixture of carbon sources during the methanol induction phase alleviates the metabolic burden derived from heterologous protein production and increases recombinant protein productivity [141,142,143]. Thus, several feeding strategies have been designed based on co-substrate utilization in *K. phaffii*, as previously reviewed [132]. For example, to avoid the transitional period prior to methanol induction, a feed strategy was established with slow enzymatic glucose feed to maximize the expression of *Rhizopus oryzae* lipase (ROL). This feed strategy maintains a low glucose concentration while avoiding cellular starvation and inactivation of pAOX1, which resulted in 3- and 6-fold higher cell density protein activity, respectively, when compared to [144].

In *Y. lipolytica*, co-substrate feeding was applied to maximize the production of recombinant human interferon (hu-IFN α2b), whose encoding gene was cloned under the control of the oleic acid inducible POX2 promoter. Initially, the cells were grown in glucose to achieve a cell density of 73 g/L. Next, the best feeding strategy consisted of continuous glucose/oleic acid feeding at a ratio of 0.02 g oleic acid/g CDW, resulting in almost twice the recombinant protein being produced [145]. In another study, a two-stage, cyclic fed-batch bioprocess was performed, aiming at increasing the productivity of the rice α-amylase SMY2. The cultivation setup consisted of the transfer of a portion of the whole fermentation broth from the growth stage to the production stage while leaving a smaller fraction of the broth for continued cell growth [146]. This resulted in twice the biomass productivity, around 1616 U·L^−1^ h^−1^ when compared to a single fed-batch fermentation process [146].

As mentioned before, in *K. lactis* recombinant genes are often cloned under the inducible promoter Lac4. Thus, cultivation strategies have involved the utilization of whey, an inexpensive waste product from the dairy industry, in batch and fed-batch fermentations. In the latter, a feed solution was added stepwise or continuously to increase biomass and yield [130,147]. Furthermore, the carbon/nitrogen ratio of the feed solution was found to be optimal at the ratio of 54:7 for the production of recombinant xylanase [130]. The galactose to glucose ratio can also improve protein production, and a ratio of 0.1 has been found to be the most effective [148].

## 6. Perspectives in Recombinant Protein Production

It is widely known that the choice of a suitable host for recombinant protein production is dependent on the protein structure and requires post-translation modifications. Biopharmaceutical recombinant proteins often rely on proteolysis, glycosylation, and correct folding to be bioactive. Furthermore, secretion is advantageous since it reduces downstream steps and increases recombinant protein yield. Within this context, utilization of yeast as hosts for heterologous protein production will become even more popular in the future. Furthermore, it is expected that the portfolio of yeast species used for this purpose will also expand with the increased availability of molecular tools that are functional in several yeast species at the same time. Also, with advances in robotics technology, the utilization of simultaneous yeast hosts in high-throughput screening will become more frequent to determine the optimal one for the desired recombinant protein. Finally, independent of the chosen yeast host, the usage of inducible promoters and fed-batch cultivation mode seems to be preferential since it is related to the highest productivity and titers of a given recombinant protein. 

## Figures and Tables

**Table 1:** Most frequent promoters used for recombinant protein production in yeasts.

Host.	Constitutive Promoters	Inducible Promoters
*S. cerevisiae*	ADH1, GAPDH, PGK1, TPI, ENO, PYK1, TEF	GAL1-10, CUP1, ADH2
*K. lactis*	PGK	LAC4, ADH4
*Y. lipolytica*	TEF, RPS7, XPR2/hp4d	POX2, POT1, ICL1
*K. phaffii*	GAP, TEF, PGK, YPT1	AOX1, FLD1, PEX8

**Table 2:** Examples of secretion factors utilized in yeasts.

Yeast	Recombinant Protein	Protein secreted	Secretion factor	Secretion Signal Source	Ref.
*K. phaffii*	EGFP	-- ^a^	HBFI	*Trichoderma reesei* Hydrophobin	[112]
*Y. lipolytica*	Invertase	--	XPR2 pre ^c^	*Y. lipolytica* Alkaline extracellular protease precursor	[113]
*Y. lipolytica*	Galactanase I	3 mg/L	XPR2 pre ^c^	*Y. lipolytica* Alkaline extracellular protease precursor	[114]
*Y. lipolytica*	α-amylase	--	XPR2 pre-pro ^c^	*Y. lipolytica* Alkaline extracellular protease precursor	[115]
*Y. lipolytica*	Aspartic proteinase II	--	Hybrid LIP2/XPR2 pre-pro ^c^	*Y. lipolytica* Alkaline extracellular protease precursor	[116]
*K. lactis*	α-amylase	0.527 U/mL	KT	Synthetic	[39]
*K. lactis*	Insulin precursor	30 mg/L	α-MF	*S. cerevisiae* α-mating factor	[117]
*K. lactis*	a-galactosidase	2 mg/L	SUC2 pre	*S. cerevisiae* invertase	[118]
*K. lactis*	Growth hormone	--	PHO5	*K. lactis* acid phosphatase	[119]
*K. lactis*	Serum albumin (HSA)	3 g/L	HSA pre-pro	-	[120]
*K. phaffii*	Horseradish peroxidase	--	α-MF	*S. cerevisiae* α-mating factor	[111]
*K. phaffii*	α1-antitrypsin	--	SUC2	*S. cerevisiae* invertase	[116]⁠
*K. phaffii*	α1-antitrypsin	--	PIR1	Proteins with internal repeats (PIR) from *K. phaffii*	[39]
*K. phaffii*	Porcine Pepsinogen	--	PHO1	*K. phaffii* acid phosphatase	[117]
*K. phaffii*	α-amylase	2.5 g/L	SUC2	*S. cerevisiae* invertase	[118]
*K. phaffii*	α-amylase	240 ug/mL	pGKL	PGKL killer protein	[51]
*K. phaffii*	EGFP	--	SCW, DSE, and EXG	Endogenous signal peptides	[121]
*K. phaffii*	EGFP	--	PIR1	Proteins with internal repeats (PIR) from *K. phaffii*	[39]
*S. cerevisiae*	β-galactosidase	0.8 ^b^	AGA2	*S. cerevisiae* Adhesion subunit of a-agglutinin	[122]
*S. cerevisiae*	β-galactosidase	0.9 ^b^	EXG	*S. cerevisiae* Exo-1,3-B-Glucanase	
*S. cerevisiae*	β-galactosidase	0.9 ^b^	α-MF	*S. cerevisiae* α-mating factor	
*S. cerevisiae*	β-galactosidase	0.9 ^b^	CRH	*S. cerevisiae* Chitin trans-glycosylase	
*S. cerevisiae*	β-galactosidase	0.65 ^b^	PLB	*S. cerevisiae* Phospholipase B	
*S. cerevisiae*	β-galactosidase	0.85 ^b^	SUN	Cell wall protein related to glucanases of *S. cerevisiae*	
*S. cerevisiae*	Hen Lysozyme	13 mg/L	α-MF	*S. cerevisiae* α-mating factor	[123]⁠
*S. cerevisiae*	Hen Lysozyme	2.6 mg/L	KILM1	*S. cerevisiae* Killer toxin type 1	
*S. cerevisiae*	Hen Lysozyme	2.1 mg/L	PHO1	*S. cerevisiae* Acid phosphatase	
*S. cerevisiae*	Hen Lysozyme	2.0 mg/L	SUC2	*S. cerevisiae* invertase	

^a^ The study did not present results with absolute values. ^b^ Relative B-galactosidase activity present in supernatant relative to the activity of the enzyme present in a strain containing a secretion factor wild-type (WT). ^c^ The pre- and pro regions indicate regions present in a sequence after gene transcription and are processed by modifications of an endopeptidase resulting in the mature protein. Secretion signals containing only the pre- region are truncated sequences lacking the pro region, which may increase or decrease the secretion of certain proteins due to the decrease in the amount of post-translational processes.
